# Study on nitrogen demand model in pakchoi (*Brassica campestris* ssp. *Chinensis* L.) based on nitrogen contents and phenotypic characteristics

**DOI:** 10.3389/fpls.2023.1111216

**Published:** 2023-02-15

**Authors:** Liying Chang, Xin Xiong, Muhammad Khalid Hameed, Danfeng Huang, Qingliang Niu

**Affiliations:** School of Agriculture and Biology, Shanghai Jiao Tong University, Shanghai, China

**Keywords:** critical nitrogen content, multi-information fusion method, nitrogen demand model, photothermal effect, pakchoi

## Abstract

**Introduction:**

In precision agriculture, the diagnosis of the nitrogen (N) nutrition status based on the plant phenotype, combined effects of soil types, various agricultural practices, and environmental factors which are essential for plant N accumulation. It helps to assess the N supply for plants at the right time and optimal amount to ensure high N use efficiency thereby reducing the N fertilizer applications to minimize environmental pollution. For this purpose, three different experiments were performed.

**Methods:**

A critical N content (Nc) model was constructed based on cumulative photothermal effect (LTF), Napplications, and cultivation systems on yield and N uptake in pakchoi.

**Results and discussion:**

According to the model, aboveground dry biomass (DW) accumulation was found equal or below to 1.5 t/ha, and the Nc value was observed at a constant of 4.78%. However, when DW accumulation exceeded 1.5 t/ha, Nc declined with the increase in DW accumulation, and the relationship between Nc and DW accumulation developed with the function Nc %=4.78 x DW-0.33. An N demand model was established based on the multi-information fusion method, which integrated multiple factors, including Nc, phenotypical indexes, temperature during the growth period, photosynthetically active radiation, and N applications. Furthermore, the model’s accuracy was verified, and the predicted N contents were found consistent with the measured values (R2 = 0.948 and RMSE = 1.96 mg/plant). At the same time, an N demand model based on N use efficiency was proposed.

**Conclusions:**

This study can provide theoretical and technical support for precise N management in pakchoi production.

## Introduction

Nitrogen (N) is an essential macronutrient for plant growth and development. N is used as a large quantity of chemical fertilizers in agricultural production including horticultural crops ranging from 40 to 350 kg N ha^-1^, and it is one of the leading environmental concerns worldwide because of its potential losses to the environment (Reis et al., 2016; Valenciana, 2017). China is the largest user of N fertilizers in the world (Yan et al., 2014). The average annual consumption of N (on an elemental basis) in China has been reported approximately 29 million tonnes (t) between 2009 and 2020, which accounted for more than 25% of the world’s annual N consumption (FAOSTAT, 2022). Excessive use of N fertilizers for optimizing crop production has resulted in a series of environmental problems (Wei et al., 2018). Furthermore, 30%–35% low N use efficiency due to excessive N applications in most cropping systems has been reported (Zhang et al., 2018). To further improve N use efficiency in different cropping systems, a rapid and accurate demand of the N status in crops is required in order to improve the N management and to achieve sustainable agricultural development goals across the world including China. Improving N use efficiency by determining the N demand status in crops is one of the most effective means of increasing crop productivity while decreasing environmental degradation caused by excessive N fertilizer use in agricultural production.

Recently, many studies have tried to enhance vegetable production and minimize environmental pollution through the N demand status in plants (Sassenrath et al., 2013; Abdallah et al., 2016; Saki et al., 2019; Du et al., 2020; Tei et al., 2020; de Paz et al., 2022). According to Min et al. (2012), the amount of N application in vegetable production is minimized by 40%, and nitrate leaching is reduced by 39.6% without changing the plant yield by optimizing the N demand in plants. N fertilization reduction from 1,200 to 600 kg N ha-1 in tomatoes, and ammonia volatilization is reduced by 37.2% without affecting the yield of tomatoes (Li et al., 2015). Different studies revealed that excessive N fertilizer applications imbalance the plant production while optimizing N demand in plants increases plant productions under different management practices in various soil and climatic conditions (Sinclair and Rufty, 2012; Sadras and Lawson, 2013; Haegele et al., 2013). The overuse of N fertilizers can be reduced by improving the N demand status model in crops. Different climatic conditions like light and temperature influence plant growth and N requirement. Thus, developing a model based on light and temperature with N applications is essential to predict the N demand status accurately in plants.

Green leafy vegetables such as pakchoi (*Brassica campestris* ssp. *Chinensis* L.) are rich source of vitamins, carotene, iron, and calcium (Hanson et al., 2009; Gupta et al., 2013). Owning to nutritional value, pakchoi has become quite popular among consumers with the rise in living standards worldwide including China (Fahey, 2015; Frede et al., 2018). Taking Shanghai as an example, the daily supply of pakchoi is over 4,000 kg, occupying a high proportion (30%–40%) of total green leafy vegetables in China (Zhu et al., 2013). Fulfilling the daily requirement of the consumers without identifying the critical N demand status in plants is considered as minimum chances due to the short growth period and complex agronomic practices like fertilization, planting season, irrigation, cultivation systems, and crop varieties (Foulkes et al., 1998; Fatemi et al., 2020; Xie et al., 2020; Hameed et al., 2022). A few studies about fertilizer and water diagnoses and simulation-based model–related strategies are conducted on green leafy vegetables (Durand et al., 2010; Errecart et al., 2014). The stimulation-based model with N fertilizer is required to identify the critical N demand status in green leafy vegetables, especially in pakchoi.

N plays a key role in dry biomass (DW) accumulation and crop yields. A suitable amount of N application is important to increase the crop yield and aboveground DW accumulation in plants (Lemaire et al., 2007; Lemaire et al., 2008; He et al., 2017; Qiang et al., 2019). Crop critical N content decreases gradually with the increase of aboveground DW accumulation (Greenwood et al., 1990; Zhao et al., 2016; Zhao et al., 2017). The relationship between the DW (t/ha) and critical N content (N_c_, %) is represented by the following allometric equation, also called as the critical N dilution curve, which is described by Lemaire and Salette (1984) for forage grasses:


(1) $${\text{N}}_{\text{c}}\left(\%\right)=a\times {\text{DW}}^{-b}$$

where *a* represents the N_c_ when aboveground DW accumulation equals 1 t/ha; *b* represents the declining rate of N_c_ with DW accumulation in the plants.

N_c_ is defined as the minimal total N contents in plants that produce maximum DW production, which is also an ideal N content throughout the plant growth period (Shan-Chao et al., 2014). The critical N dilution curves are used to determine plants" N requirements and to calculate the N nutrition index (NNI) that quantifies the N status in the plants (Zhao et al., 2016; Zhao et al., 2017; Qiang et al., 2019; Ciampitti et al., 2022). The NNI is used as a quantifier in dynamic models to access the N fertilizers" efficiency on growth and yield (Zhao et al., 2016; Zhao et al., 2017; Ciampitti et al., 2022). Previously, critical N dilution curve models are constructed for different crops including melons (Fogaça et al., 2008), tomatoes (Wang et al., 2013), rice (He et al., 2017), barley (Sedlář et al., 2017), maize (Zhao et al., 2017), wheat (Yin et al., 2018), and carrots (Shlevin et al., 2018), among other crops. According to the critical N dilution curves of crops, the NNI is defined as the ratio of actual N content to the N_c_ of the aboveground crop part, which is used for quantifying the N status in plants. According to Justes et al. (1997), the NNI equation is as follows:


(2) $$\text{NNI}={\text{\%N}}_{\text{a}}/\%{\text{N}}_{\text{c}}$$

where %N_a_ represents the actual N content and %N_c_ represents the critical N content corresponding to the actual biomass of the crop. The crop N nutrition status is optimal if the NNI is equal to 1; if it is <1 and >1, then it is in excess and deficient, respectively (Lemaire et al., 2008). The NNI is used to evaluate the crop N status based on critical N dilution curves and yields in growing crops.

For high plant yield, the use efficiency of the N fertilizer is required to increase the growth and productivity of the crops in the growing season (Zhao et al., 2017). In N fertilizer management practices, it is a big challenge to balance the demand and supply of N due to the complexity of the N cycle in the soil for plant growth and production under different growing and climatic conditions. Effective N regulation/management during crop growth is a key for efficient crop production, improving crop yield and quality (Zhao et al., 2017).

Different sensor technologies and corresponding models are used to calculate the actual supply and utilization of N in the real-time crop N status under different soil types and agricultural practices for various crops (Zhao et al., 2005; Zhao et al., 2016; Ye et al., 2021; Yu et al., 2022). Additionally, the N fertilizer is a macronutrient to be applied in a specific amount and rate and according to the requirements of the plants in conventional practices (Tei et al., 2020). Furthermore, high N fertilizer use efficiency can be increased by applying N fertilizers efficiently to fulfill the demand for crops (Yousaf et al., 2017). N demand in crops is based on the crop types; DW production; phenotypical traits of crops (Xiong et al., 2019); effects of soil types; management practices; and environmental factors such as temperature, photosynthetically active radiation, and water (Zhao et al., 2005; Zhou et al., 2019; Chang et al., 2021). In most cases, the effect of different fertilizer applications on plant biomass and field crop production has been evaluated. However, an effective method for quantifying the N demand status in vegetables, specifically pakchoi, has not yet been developed. The development of a plant-based diagnostic tool is required for a better understanding of the pakchoi N demand status under different growing conditions. The objective of this study was to determine the critical N dilution curve for pakchoi during growth in different soil types based on N_c_ and the phenological characteristics of pakchoi and to validate it in different climatic conditions.

In this study, we conducted different experiments with different N levels to assess the growth status (phenotypic characteristics and biomass accumulation) and N nutrition status of pakchoi under different soil types and photothermal environmental factors.

## Material and methods

Three experiments were conducted based on different phenotypic characteristics, soil, and climatic conditions under different N levels in a greenhouse at the Agricultural Engineering Training Center, Shanghai Jiao Tong University, Shanghai, China. The pakchoi cultivar “Huawang” was chosen for the experiments. An overview of pakchoi growing experiments in a greenhouse is shown in **Supplementary Figure S1**.

### Experiment 1

The experiment was conducted from September to October 2016. Each experimental pot contained dimensions of 13.5 cm length × 11 cm width × 8.9 cm height. Each pot was filled with 500 g of dry fluvoaquic soil. The physicochemical characteristics of fluvoaquic soil were as follows: an organic matter of 39.73 g/kg, total N 1.43 g/kg, available phosphorus (P) 23.31 g/kg, available potassium (K) 136 mg/kg, nitrate-N 13.3 mg/kg, ammonium-N 8.5 mg/kg, and a soil pH of 8.29. Four different N levels were used as follows: 0 (no urea applied, N_0_), 0.05 g N/kg soil (54 mg urea in a pot, N_0.05_), 0.1 g N/kg soil (107 mg urea in a pot, N_0.1_), and 0.2 g N/kg soil (214 mg urea in a pot, N_0.2_). In total, 180 pots were prepared for the experiment, and 45 pots were used for each treatment. A total of 0.3291 g of KH_2_PO_4_ was used as K and P sources in each pot, which contained K = 0.189 g/kg and P = 0.15 g/kg. Urea and KH_2_PO_4_ fertilizers were used at the sowing time as a basal dose. There were 10 healthy seeds sown in each pot and thinned to one healthy plant at the two-leaf stage. Soil moisture was maintained at 75% ± 5% through weighting. Plants were harvested on the 11th, 18th, 31st, 38th, and 45th days after sowing. At each sampling time from each treatment, nine uniform plants were chosen for fresh biomass (FW), DW, and N content evaluation.

### Experiment 2

The experiment was carried out from December 2017 to February 2018. Each pot was 14.5 cm × 10.5 cm × 12.0 cm in size and contained 1.5 L of cultivation substrates. The chemical properties of the substrate contained organic matter 270.3 g/kg, total N 5.3 g/kg, available P 1.24 mg/kg, available K 118 mg/kg, and available N 332 mg/kg, and pH was 6.77. The seeds were sown in a plug tray and transplanted to the pots at the two-leaves stage. Each pot contained one plant. N treatments were applied to the seedlings 1 week later. N treatments were applied as follows: 0 (N_0_), 0.134 (N_1_), 0.163 (N_2_), and 0.191 (N_3_) g N/pot. Urea was used as an N source and applied in six split doses. A total of 0.461 g of KH_2_PO_4_ is used as K and P sources, containing 0.189 and 0.15 g, respectively, in each pot and applied once at the beginning of N treatments. Each treatment contained 60 plants, and 240 plants were sown in total. The experiment was continued for 42 days. After N treatments, 12 uniform plants were collected every 7th day from each treatment for data analysis.

### Experiment 3

The experiment was conducted from October to December 2018. Each experimental pot was 12.0 cm × 8.8 cm × 10.8 cm in size and contained 1.5 L of cultivation substrates. The pH of the substrate was 6.46 and contained available N of 163 mg/kg, available P of 99 mg/kg, and available K of 125 mg/kg. A similar methodology as mentioned in experiment 2 was followed for seeds grown and transferred to the pots. Urea (0.15 g N kg^-1^ in the substrate) and KH_2_PO_4_ (P = 0.15 g kg^-1^ in the substrate and K = 0.189 g kg^-1^ in the substrate) were applied along with irrigation. One-third of the fertilizers were applied after the 5th day of transplanting, and two-thirds were applied after the 10th day. After N treatments, 20 plants were used to take image analysis data, and 9 plants were used to measure N content every 5th day. Image acquisition and analysis were determined as described by Xiong et al. (2019) and Chang et al. (2021).

Both experiments (1 and 2) were conducted to estimate the relationship between DW and N_c_ with a relative growth rate (day^-1^) under different N applications. Experiment 3 was used to estimate the relationship between phenotypic characteristics with N_c_. The results of all three experiments were used together to estimate the N_c_ with DW under N application as predicted by experiment 3 and developed a model based on the results of all three experiments.

### Measurement of plant fresh, dry biomass, and nitrogen content

The electronic balance was used to determine the FW and DW of plants. The samples were put at 80°C for 24 h to obtain the plants DW. A 60-mm sieve was used to grind and filter the dried plants. A 5 g ground sample was taken for N content (g kg^-1^) measurement with Vario ELIII (Elementar, Germany) (Justes et al., 1994).

### Environmental factors

In all three experiments, temperature and photosynthetically active radiation in the greenhouse were recorded every 5 min with a portable automatic weather station (HOBO-U30, Onset, Bourne, MA, USA) for the whole growing season. The average environmental factor value of every 1 h was recorded. The average meteorological data were measured by taking the average values of 24 h and used as average data for 1 day (**Supplementary Figure S2**). The same procedure was repeated for the whole growing season of all three experiments to obtain the average value of meteorological data (**Supplementary Table 1**).

### Calculation of photothermal effect

As explained by Zhou et al. (2019) and Justes et al. (1994), the photothermal effect (LTF) was used to evaluate the effects of temperature and radiation on yield, DW accumulation, and N content in the aboveground parts of pakchoi.

### Nitrogen content model

The cropping systems simulation (CropSyst) model is a general crop growth model and employs the indexes of maximum N content (N_max_), critical N content (N_c_), and a minimum of N content (N_min_) in the N content curve (Stöckle et al., 2003; Nunn et al., 2017). In addition, the N_max_ is added at the early growth stage (Stockle et al., 1994; Stockle and Debaeke, 1997; Ekbladh and Witter, 2010). The following three characteristics of N content curves can be obtained by the following equations for pakchoi:


(3) $$N_{max}=min\left(N_{max},\;\;a_{max}\times DW^{-b}\right)$$

(4) $$N_{c}=min\left(0.7N_{max},\;\;a_{c}\times DW^{-b}\right)$$

(5) $$N_{min}=min\left(0.4N_{max},\;\;a_{min}\times DW^{-b}\right)$$

where DW (t/ha) represents the biomass of crops under suitable environments by using Equation 1 (Lemaire et al., 2008), the coefficient ‘*a*’ is the value of N_c_ when DW value is 1 t ha^-1^, and the exponent ‘*b*’ determines the rate of decline in N_c_ such as the slope of the relationship between ln(N_c_) and ln(DW) in **Supplementary Figure S3** (Stockle and Nelson, 1996). The following parameters were determined as described by Ekbladh and Witter (2010): *a*_max_= *N*_max_/(2^-b^), *a*_c_ = 0.7 × . *N*_x_/1.5^-b^, *a*_min_= 0.4 × . *N*_x_/0.5^-b^, and *b* = 0.33 was used for pakchoi.

### Model construction and verification

Plant FW and the corresponding N_c_ were calculated according to the cumulative photothermal effect from the second experiment. The N_c_ was based on phenotypical parameters, and a random forest model was used for crop N demand (N_Demand_) prediction in all experimental data. The N_Demand_ is defined as the necessary N amount for maintaining the maximum growth rate and the maximum biomass accumulation of a crop and depends on crop growth vigor and N_c_ (Greenwood et al., 1990; Qiang et al., 2019). Through destructive sampling, the FW, N_c_, and N_a_ contents were used to calculate the actual *N*_Demand_ of crops. The root mean square error (RMSE) and the coefficient of determination (*R* ^2^) were used to determine the N demand models in all experiments as described by Xiong et al. (2019).

### Nitrogen demand and supply model

Based on dynamic management of the critical N curve and the plant phenotype, the real-time crop demand for nutrients was monitored, which was used as conducive for changing the current situation of all applied N fertilizers as basal doses and reducing the N losses to the environment. Theoretically, crop N demand refers to the N difference between N_c_ and N_a_ (Bo et al., 2021). However, crop N accumulation also depends on environmental factors and agricultural practices other than N demand. When N supply is applied more than the demand, crops can absorb enough N from the soil. Therefore, N demand and supply models are shown in the following equations:


(6) $${\text{N}}_{\text{Demand}}=0.39\times \text{FW}\times \left(N_{c}-N_{a}\right)$$

(7) $${\text{N}}_{\text{Supply}}=0.39\times \text{FW}\times \left(N_{c}-N_{a}\right)/N_{Use\;efficiency}$$

where N_Demand_ (g) refers to crop demand for N; 0.39 is the crop coefficient; N_Supply_ refers to the N amount (g) that was supplied; FW (g plant^-1^) represents aboveground FW; and N_c_ (%) is the critical N content, and N_a_ (%) is the actual N content.

Yield (fresh biomass) model based on photothermal effect


(8) $$\text{FW}=ae^{\left(b\times LTF\right)}\text{= }0.2816\times e^{\left(0.121\times LTF\right)}$$

where *a =* 0.2816* g* plant^-1^, the aboveground FW of 18-day-old seedlings from the second experiment and *b =* 0.121; the maximum DW accumulation rate was changed with the LTF. The LTF was calculated as described by Zhou et al. (2019):

1) Temperature (thermal) effect


(9) $${\text{f}}_{\text{T}}=\{\begin{array}{c} \;\;0\;\;\;\;\;\;\;\;\;\;\;\;\;\;\;\;\;\;\;\;T\leq {\text{T}}_{\text{b}}\;or\;T\geq {\text{T}}_{\text{m}}\\ \left(\text{T}-{\text{T}}_{\text{b}}\right)/\left({\text{T}}_{0}-{\text{T}}_{\text{b}}\right){\text{ T}}_{\text{b}}<T<{\text{T}}_{0}\\ 1\;\;\;\;\;\;\;\;\;\;\;\;\;\;\;\;\;\;\;\;\;\;\;\;\;\;\;\;T={\text{T}}_{0}\\ \left({\text{T}}_{\text{m}}-\text{T}\right)/\left({\text{T}}_{\text{m}}-{\text{T}}_{0}\right){\text{ T}}_{0}<T<{\text{T}}_{\text{m}} \end{array}$$

where *f*_T_ is the relative temperature effect at T (°); T_0_ is the optimal growth temperature of 24°; T_b_ represents the minimum growth temperature (7°); T_m_ represents the maximum growth temperature (35°); and T (°) indicates the average temperature in the *i*th hour from the second experiment.

2) Light effect


(10) $$f_{I}=\left(1-e^{-\text{α}I}\right)$$

where I (mmol·m^-2^·d^-1^) indicates relative daily photothermal product; α is the curvature of function, and the value is 0.001.

3) Cumulative photothermal effect (LTF) during crop growth


(11) $$\text{LTF}=\sum^{}\left(f_{T\left(j\right)}\times f_{I\left(j\right)}\right)$$

where *f*_I(_ *_j_*_)_ and *f*_T(_ *_j_*_)_ indicate the light and thermal effects, respectively, at the *j*th growth day for the second experiment.

### Model development and validation

The data from experiment 2 was used to validate the LTF-based FW formation model, DW, and N accumulation model. The relative standard error (RE) is used as the evaluation factor for the degree of agreement between the simulated and measured values. The calculation method is shown in Equation 12. A linear regression equation was used to compare the simulated and measured values, and the coefficient of determination *R^2^* of the linear regression equation was determined by the *t*-test.

### Correlation significance test


(12) Relative standard error of prediction (%)= (SE of regression estimated value/measured value) × 100

### Critical nitrogen content curve

The following equation was established based on the above experiments:


(13) $${\text{N}}_{c}\%=\{\begin{array}{c} 4.78\;\;\;\;\;\;\;\;\;\;\;\;\;\;\;\;\;\;\;\;\;\;\;\;DW\leq 1.5t/ha\\ 4.78\times DW^{-0.33}\;\;\;\;\;\;\;\;\;\;\;\;\;DW>1.5\;t/ha \end{array}$$

(14) FW=25.64×DW

Where N_*c*_ . %epresents the critical N content, DW (g plant^-1^) and FW (g plant^-1^) indicate plant dry and fresh biomass (FW), respectively. If FW ≤ 38.64 t/ha, then N_c_ = 4.78%. If FW > 38.64 t/ha, then N_c_ declines with increasing FW in pakchoi.

### Data analysis

The N_c_ contents were analyzed as described by Justes et al. (1994). The analysis of variance (ANOVA) was used to compare the aboveground DW under different N applications and the corresponding parameters at each sampling date and under LTF using the SPSS 26.0 version software (SPSS Inc., Chicago, IL, USA). Duncan’s 95% significance level test was used to evaluate the significance of the difference between the treatment means of each measured parameter in all experiments. Origin Pro 9.0 was used for data visualization.

## Results

### Critical nitrogen content model on the basis of dry biomass and aboveground nitrogen content

**Figure 1:**
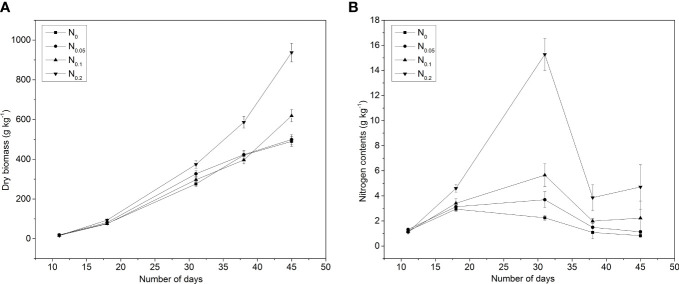
Effects of nitrogen (N) application on **(A)** plant dry biomass (DW) and **(B)** N contents under different intervals of time in pakchoi (Experiment 1). N_0_ = 0 g N kg^-1^ soil, N_0.05_ = 0.05 g N kg^-1^ soil, N_0.1_ = 0.1 g N kg^-1^ soil, and N_0.2_ = 0.2 g N kg^-1^ soil.

N application showed a significant effect on the aboveground DW of pakchoi throughout the growth period, ranging from the minimum value of 18.5 mg per plant for N_0_ to 937.23 mg per plant for N_0.2_ in experiment 1 (**Figure 1A**). The aboveground DW showed an increasing trend with changing N levels in all treatments at different intervals. However, N applications did not show a significant difference in the early growth days (11th day). Additionally, treatment N_0.2_ significantly increased the DW relative to other treatments on the 31st, 38th, and 45th days after sowing. However, there was no significant difference between N_0_ and N_0.05_ treatments for the DW of pakchoi at the 45th day. The DW in treatment N_0.2_ was approximately 23.68%, 35.51%, 39.43%, and 91.22% higher compared to N_0_.

The aboveground N content of pakchoi on DW basis was shown within the range of 1.6%–7% during the whole growth period under all applied N applications in different growing days (**Figure 1B**). For a particular time, aboveground N content showed an increasing trend with the increase of N levels. The increasing trend in aboveground N content was found approximately 5.83%–6.75%, 0.18%–11.74%, 32.76%–142.24%, 28.81%–139.55%, and 1.88%–96.88% more in N applications (N_0.05_, N_0.1_, and N_0.2_) as compared to N_0_ on the 18th, 31st, 38th, and 45th days after transplanting, respectively. After the 31st day, the aboveground N content decreased with increasing pakchoi growth, and it decreased from 6.52% to1.60%, 6.93% to 1.63%, 6.96% to 2.33%, and 6.90% to 3.15% in N_0_, N_0.05_, N_0.1_, and N_0.2_, respectively.

**Figure 2:**
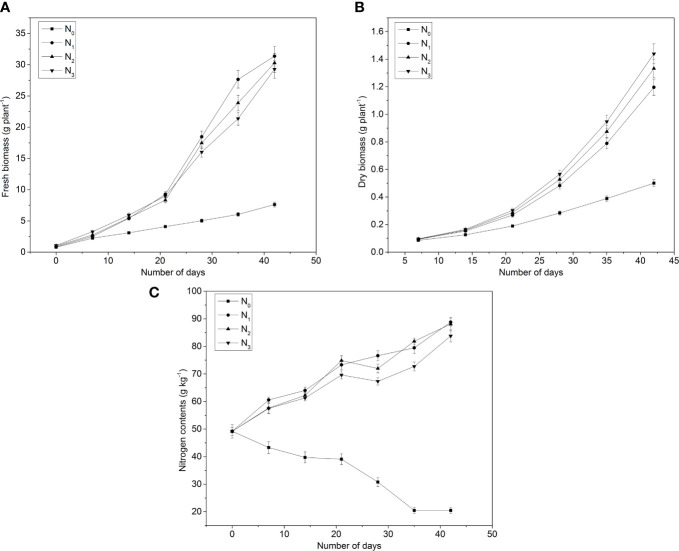
Effects of N application on **(A)** plant fresh biomass (FW), **(B)** plant DW, and **(C)** N contents under different intervals of time in pakchoi (Experiment 2). N_0 =_ 0_ g_ N pot^-1^, N_1 =_ 0.134_ g_ N pot^-1^, N_2 =_ 0.163_ g_ N pot^-1^, and N_3 =_ 0.191_ g_ N pot^-1^.

In experiment 2, the accumulation of FW, DW, and N content in the shoots of the pakchoi was measured (**Figure 2**). The aboveground FW of pakchoi significantly improved by all applied N levels as compared to control (**Figure 2A**). The FW increased continuously with N application in the whole growth period of pakchoi. The maximum aboveground FW of pakchoi was found by N_1_ (31.36 g per plant) as compared to other applied N levels after 42 days. There was no difference in the FW of pakchoi by applied N treatments in the early stage (7–21 days). On the 35th day, N_1_ showed a significant difference in the FW of pakchoi as compared to other applied N applications. However, all applied N treatments showed a significant difference in the FW of pakchoi as compared to control from the 14th to 42nd days. However, pakchoi contained low FW with a higher N level in N_3_ as compared to other applied N levels.

The aboveground DW of pakchoi did not show a significant difference in early growth (7th and 14th days). However, at a later stage, treatment N_3_ (1.43 g per plant) showed a significant difference in the aboveground DW of pakchoi as compared to other applied treatments after the 42nd day. The DW increased continuously with increasing the plant growth cycle. However, DW was regulated with applied N levels. During the 7th–28th day after treatments, the N_3_-treated pakchoi accumulated the highest DW (1.43 g per plant). All applied N treatments showed a significant difference as compared to N_0_ (**Figure 2B**).

The response of the plant aboveground N content under different N applications was observed after the seventh day in experiment 2 (**Figure 2C**). However, the aboveground N content did not exhibit a significant difference among the applied N treatments except N_0_ until the 28th day, and it was found higher in the whole growing season as compared to N_0_. On the 28th day, the aboveground N content was positively associated with the N application rate. On the 35th and 42nd days after transplanting, the aboveground N content did not show a significant difference between N_1_ and N_2_. However, it showed higher N contents as compared to N_3_.

**Figure 3:**
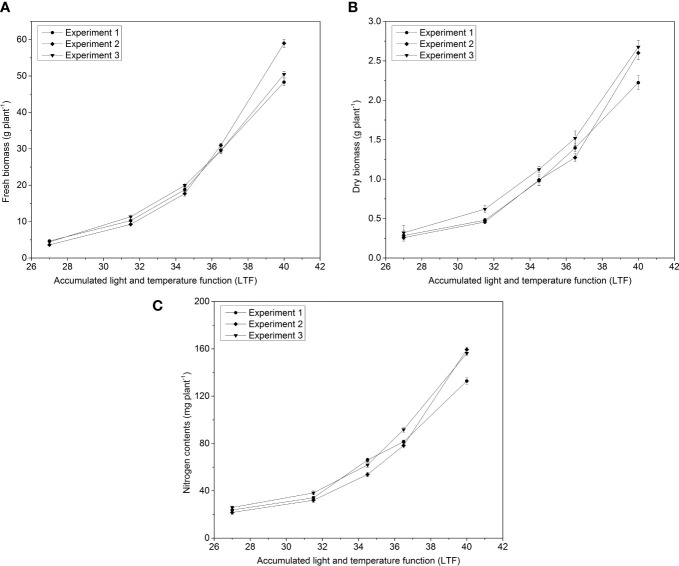
The changes of **(A)** plant FW, **(B)** plant DW, and **(C)** N contents with changing LTF in pakchoi under different N applications in three experiments.

All the aboveground FW and DW of pakchoi were affected by LTF in all experiments (**Figures 3A, B**). Aboveground FW and DW exponentially increased with increasing LTF. In experiment 2, the aboveground FW (59.00 g) was influenced more than experiment 1 and 3 (**Figure 3A**). However, aboveground DW were found more influence and the maximum value was observed (2.67 g plant^-1^) in experiment 3 as compared to experiment 1 (2.22 g) and experiment 2 (2.59 g) along with LTF under all applied N treatments (**Figure 3B**). Maximum N contents were observed in experiment 2 (159.42 g kg^-1^) and experiment 3 (156.31 g kg^-1^) as compared to experiment 1 (32.82 g kg^-1^), under changing the LTF values in the pakchoi- growing season (**Figure 3C**).

### Aboveground nitrogen content

#### Photothermal effect

From the meteorological data in each experimental pakchoi growth period (**Supplementary data Table 1**), it showed that the meteorological conditions of the 1st and 3rd experiments were found similar to each other. The temperature and photosynthetically active radiation were found higher in the second experiment, ranging from 19°C to 31°C (**Supplementary Figure S2A**). The daily average air temperature of all three experimental periods was shown (**Supplementary Figure S2**). The variation in average temperature was found relatively high in experiment 2, and the maximum differences in temperature were observed 10.1°C, 11.2°C, and 9.2°C in experiment 1, 2, and 3, respectively. Similar daily average temperature values of the first and third experiment was found. Sometimes, temperature fluctuated at 13°C–23°C, and the average temperature value during the growth period was observed approximately 19°C. The low value of daily average temperature was found, which was not conducive to pakchoi growth. The average daily temperature value in the second experiment was found between 19°C–31°C, which showed the optimum growth rate for pakchoi. Within the temperature range, the average temperature value during the growth period was observed 26.3°C, slightly higher as compared to the first experimental period. Effective sunlight radiation measurements during all three experiments (**Supplementary Figure S2**) showed that the effective sunlight radiation fluctuated widely in all three growing seasons. The maximum variation in values was found approximately 9.4, 14.8, and 9.3 mol/m^2^ in experiments 1, 2, and 3, respectively. The mean value of solar radiation in the first and third experiments was found approximately 5.3 mol/m^2^, which was observed significantly lower than the value of effective sunlight radiation in the second experiment (9.5 mol/m^2^) (**Supplementary Figure S2B**).

**Figure 4:**
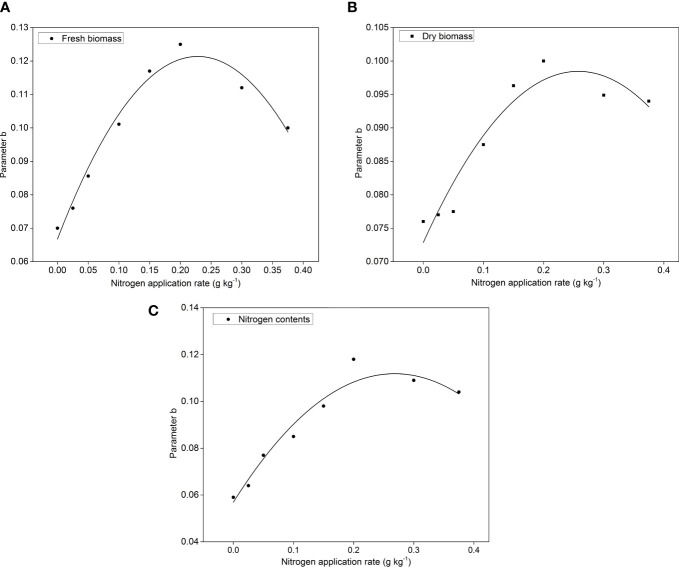
The relationship between parameter b and N application rate for **(A)** FW, **(B)** Dry DW and **(C)** Nitrogen contents in pakxhoi with the following formula N_c_(*%*)=*a*×DW^−^_*b*_ .

**Figure 5:**
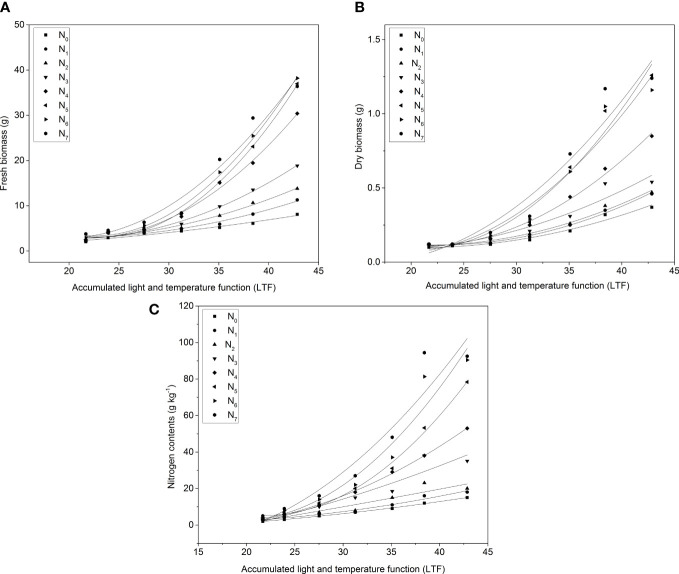
The changes of **(A)** FW, **(B)** DW, and **(C)** N contents with changing LTF under different N application rates. N_0_ = 0, N_1 =_ 0.05, N_2 =_ 0.1, N_3 =_ 0.134, N_4 =_ 0.15, N_5 =_ 0.163, N_6 =_ 0.191, and N_7 =_ 0.2_ g_ pot^-1^.

The temperature and photosynthetically active radiation data in the glass greenhouse were used in three experiments to estimate the total growth of pakchoi. The LTF of pakchoi was compared with the yield, DW, and N uptake in three experiments (**Figure 3**). The model was fitted to determine the model yield and DW content of pakchoi under different N applications in experiment 2 and the N accumulation and LTF to determine the relationship between N applications and model parameters were shown (**Figure 4**). The FW, DW, and N contents of pakchoi within the model under different N applications and LTF were used to evaluate the data validation of all experiments (**Figure 5**).

### Model description

In all three experiments, after transplanting the FW, DW, and N contents increased in pakchoi under different N applications with LTF (**Figure 3**). The biomass accumulation rate and N uptake were also increased due to variation in climatic conditions. DW accumulation (**Figure 3B**) and N uptake (**Figure 3C**) in experiment 3 were consistent throughout the growth period with LTF. Experiment 2 showed higher DW accumulation as compared to other experiments. Experiment 3 did not show a significant difference until the harvest day in FW accumulation (**Figure 3A**). However, all experiments showed better results with LTF for FW, DW, and N accumulation under different N applications.

All parameters were exponentially related to the LTF: Y = *a* × EXP(*b* × LTF), where LTF is the cumulative photothermal effect. Parameter *a* is the FW of the plant at the time of transplanting (g plant^-1^), DW (g plant^-1^), and N uptake value (mg plant^-1^), while parameter *b* represents FW, DW, and N contents with LTF. The F values ​​of the three models for FW, DW, and N contents under different N applications were shown to be significantly different, and the *R^2^* was higher than 0.9 (**Table 1**).

**Table 1:** Relationship between fresh biomass, dry biomass, nitrogen (N) contents, and the cumulative photothermal effect in experiments 1, 2, and 3 under N application rates in pakchoi.

Experiment No.	Fresh biomass accumulation model		Dry-weight accumulation model		Nitrogen uptake model	
	Adj R^2^	F value	Adj R^2^	F value	Adj R^2^	F value
Exp 1	0.9950	799.76*	0.9770	170.79*	0.9811	208.84*
Exp 2	0.9968	1251.78*	0.9949	784.49*	0.9942	685.62*
Exp 3	0.9976	1666.55*	0.9957	924.48*	0.9980	1980.86*

*represents the significant difference at ≤ 0.01.

The amount of N application rates significantly affected the FW, DW, and N contents of pakchoi under different N levels. The FW, DW, and N uptake with LTF still fit an exponential function (**Figure 3**). When LTF is less than 38, aboveground FW, DW, and N contents all increased with the increase of N application rate when it was greater than 38. N_1_, N_2,_ and N_3_ did not show a significant difference in the FW and DW of pakchoi among the three treatments from the first and second experiments. However, the N accumulation in the pakchoi increased continuously. In addition, the FW, DW, and N contents of pakchoi under different N application levels were established, respectively.

The cumulative model of thermal effect and the relationship between model parameter *b* and N application rates were analyzed. The results showed that, at the tested N levels, the parameters *b* of the FW, DW, and N contents model all increased and then decreased with the increase of the N application rates (**Figures 4**, **5**). In the model, the parameter *b* was significantly affected by the amount of N applied and had a quadratic function (F) relationship with the amount of N applied (g/kg): FW model parameter *b*=0.0665+0.4749*F*-1.0323*F^2^*, *R^2 =^* 0.9649; DW model parameter *b*=0.0729+0.2052*F*-0.3986*F^2^*, *R^2 =^* 0.9026; N content model parameter *b*=0.0599+0.4114*F*-0.8141*F^2^*, *R^2 =^* 0.8588. The amount of N applied was found at 0.2 g/kg; the three models’ parameter *b* reached the maximum value; and the FW, DW, and N content models showed a maximum value. The maximum values ​​of parameter *b* were found approximately 0.121, 0.099, and 0.112 in FW, DW, and N contents in experiments 1, 2, and 3, respectively. The cumulative rate of the FW and DW of pakchoi and N content was first increased and then decreased with the increase of the N application rate. The N application rate was 0.2 g N kg^-1^ in the substrate; pakchoi showed the highest growth rate and N contents (**Figure 5**).

**Figure 6:**
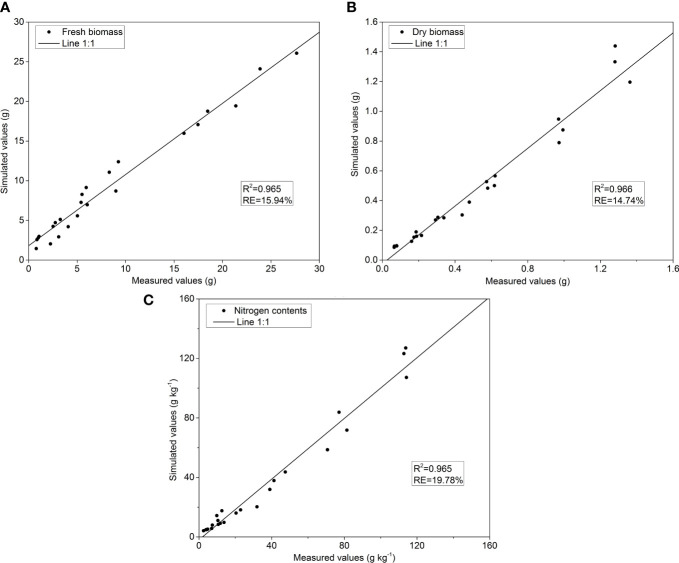
The relationship between measured values (x-axis) and simulated values (y-axis) for **(A)** FW **(B)** DW and **(C)** N contents under different N application rates in pakchoi. R_2_= the correlation coefficient of simulated and measured values and RE = relative standard error for simulated and measured values (Equation 12).

The simulated values of aboveground FW, DW, and N contents in pakchoi from the data of experiment 3 were exponentially increased with increase in the pakchoi growth period (**Figure 6**). The data from experiment 2 were used to verify the yield, DW accumulation, and N contents for the simulation model; the 1:1 linear relationship between the simulated values and the observed values were shown (**Figure 6A–C**). The relationship between simulated and measured values for FW was found approximately r=0.946, R^2 =^ 0.965 and RE = 15.94%, for DW was found about r=0.940, R^2 =^ 0.966 and RE = 14.74%, and for N contents was found about r=0.970, R^2 =^ 0.965 and RE = 19.78%, respectively (**Figure 6**). It showed that the pakchoi production based on the photothermal effect on the aboveground FW, DW, and N uptake was predicted more accurately by the model of biomass accumulation and N uptake.

### Critical nitrogen content

**Figure 7:**
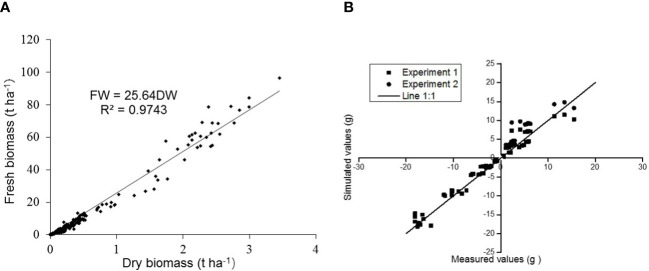
**(A)** The relationship between FW and DW under different N application rates of three experiments in pakchoi and **(B)** measured (x-axis) and simulated (y-axis) values of aboveground pakchoi biomass for the validation of the N requirement model. R^2^ = the correlation coefficient of FW and the DW values and FW calculated by using Equation 13.

**Figure 8:**
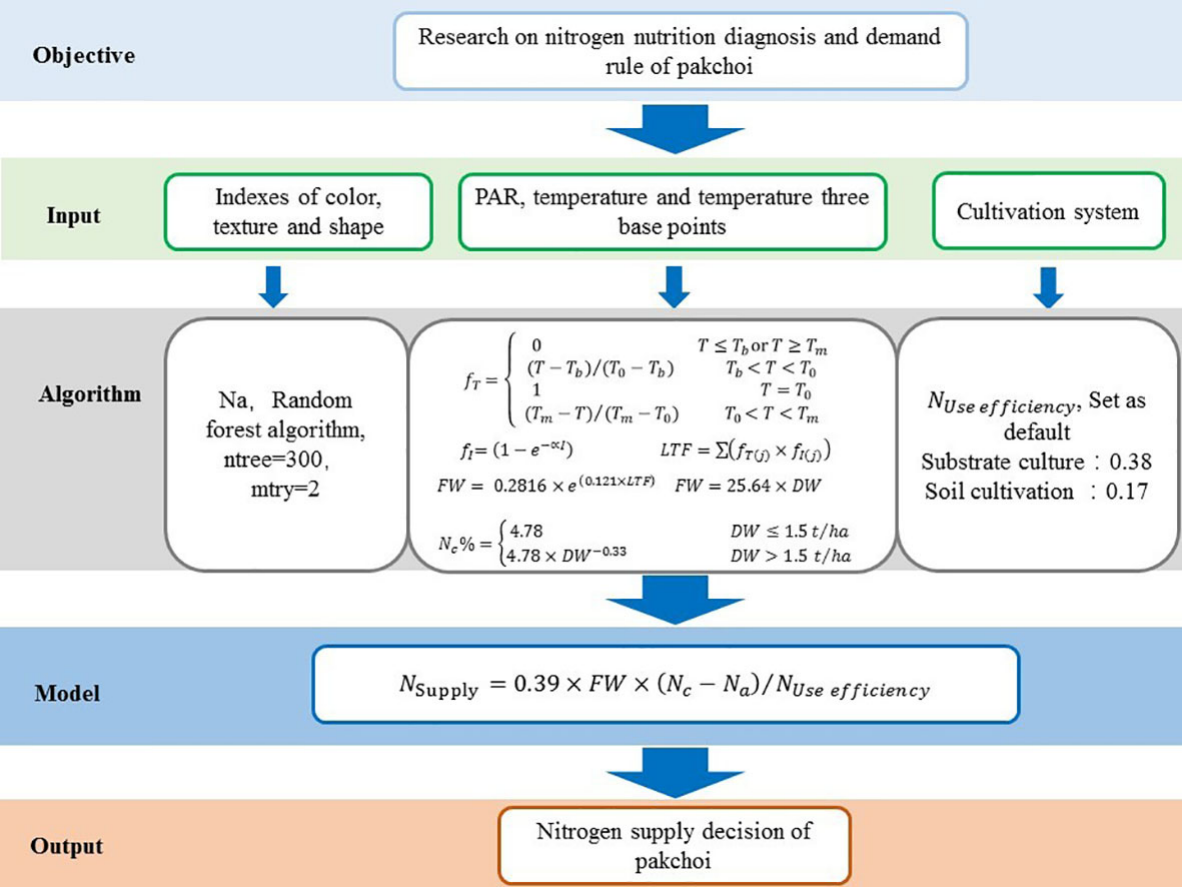
An overview of the N demand status model based on three experiments under different N applications in pakchoi.

The aboveground N content from experiment 1 was approximately 6.52%–6.96% on the 11th day after sowing when the two cotyledons of pakchoi were fully expanded. Taking the average N content (6.83%) of the four different N applications on the 11th day after sowing as the maximum content (*N*_max_), the N content curve was established based on the CropSyst model (**Figures 7**, **8**). DW was below 1.5 t/ha; the critical aboveground N content was constant at 4.78% (**Figure 8**). However, DW was observed above 1.5 t/ha; the critical aboveground N contents began to decline by following the equation   Nc %=4.78×DW^−0.33^ .he N supply satisfied the N_c_ of crops throughout the whole growth period, and the crop grew at the highest rate. In our study, the aboveground N content was found 5.45% for N_0_ on the 18th day of old seedlings, which was observed higher than the critical N content. In comparison, it was observed approximately 2.32% on the 31st day of old seedlings (three-leaf stage), which was found significantly lower than the N_c_. The results revealed that soil N applications were found adequate for the growth of pakchoi before the three-leaf stage, and the N fertilizer should be applied later.

### Nitrogen use efficiency

Using the critical N_c_ curve in Equation 13, N use efficiencies in substrate-grown and soil-grown pakchoi were found 38% and 17%, respectively.

### Verification of nitrogen demand model

In the verification of the N demand model, the results showed that predicted N demand determined accurately with actual N content, with *R^2^* = 0.948 and *RMSE* = 1.96 mg plant^-1^ (**Figures 7A, B**). The N demand model was developed based on multiparameter fusion including phenotypic traits to predict the crop N contents accurately (**Supplementary Tables 2, 3**). The validation of the N requirement model with simulated and measured pakchoi values was obtained (**Figures 7B**). An overview of the N demand model for pakchoi was established (**Figures 8**).

## Discussion

### Reasons for plant nitrogen dilution

Pakchoi is an important vegetable with great economic value. It has attained a pronounced attention of plant scientists to modulate for increasing productivity. The application of N fertilizers has shown a significant impact for increasing the growth and yield of pakchoi (Xiong et al., 2019). However, economical use of the N fertilizer is required for optimum plant growth under different agroclimatic conditions. The N fertilizer is dominant in crop growth and quality (Anas et al., 2020). N application increased DW ranging from 0.01 to 0.937, 0.08 to 1.43, and 0.25to 2.6 g plant^-1^ in experiments 1, 2, and 3 respectively. Taking into account all the data from three experiments in the present study, increasing the N applications increased the plant biomass production in pakchoi at a certain point. After that, it showed a decline in N contents and DW production with an increase in N supply and did not show more plant biomass accumulation in pakchoi (**Figure 1**). During deficiency, sufficient N application is used to improve the plant growth and yield (Min et al., 2012). Similar trends have been reported for white cabbage (Ekbladh and Witter, 2010) and wheat (Sinclair and Rufty, 2012). A suitable N level is beneficial to uptake the nutrients from soil solutions to improve the N contents and yield in plants (Bassirirad, 2000; Sun et al., 2020). In pakchoi, N applications have increased the N contents and the N dilution curve fits into the Michaeli–Menten equation (Xiaochuang et al., 2015). In our study, the FW, DW accumulation rate, and N uptake rate (*b*) were increased with the increase of the N application rate in the range of 0–0.2 g kg^-1^ soil. The results showed that N contents changed with changing the N application rates, which showed consistency with measured values in all experiments and based on this kinetic N ion uptake by pakchoi to predict the FW model in pakchoi. However, N supply was found up to 0.3 or 0.375 g kg^-1^ soil, and the FW, DW accumulation rate, and N uptake rate did not increase with increasing N application. Increasing N application decreases the N contents at a certain point and causes a decline in the yields of horticultural crops (Zhao et al., 2022).

N_c_ can be applied to the dynamic management of crop N. The daily demand for crop N is the amount of N applied per day to maintain the potential growth rate of the crop. N is provided according to the plant demands to meet the N uptake rate of the crop in the same manner as the rate of dry matter accumulation. Based on this, the dynamic N management method was initially applied to leafy plants, such as grass crops and alfalfa and later extended to wheat, corn, rapeseed, and other crops (He et al., 2017; Shlevin et al., 2018). However, the rate of accumulation and N uptake of crop dry matter is often not constant, which is affected by factors such as growth period, the cultivation medium, temperature, and light (Zhao et al., 2017; Yin et al., 2018). In our study, growth period and cultivation conditions significantly influenced DW and N contents in pakchoi under different N applications and increased DW at a certain limit. After that, it decreased with increasing the N application level in pakchoi for precision agriculture.

Crop growth models can provide evidence for the optimization and precise management of different crops under different cultivation conditions. The NNI has been calculated on the basis of the N_c_ dilution curve, which can be used for diagnosing the status of the crop nutrition, i.e., deficient, optimum, or maximum consumption of N nutrition (**Supplementary Figure S3**). The present study showed that the pakchoi yield was found greater than 38.4 t ha^-1^ in three experiments and significantly affected under the N applications, growing conditions, and plant development stages (**Table 1**). In the present study, experimental observations and simulation studies were combined, and the FW, DW, and N accumulation model of pakchoi was established based on the variation of the FW, DW, and N uptake of pakchoi. The results showed that the simulated model contained a good descriptive and predictive impact on FW, DW, accumulation and N contents in pakchoi. Stimulated and measured values correlated with each other based on N applications along with LTF for FW, DW production, and ultimate N contents in pakchoi. Different temperature and radiation-driven crop models have been constructed (Liu et al., 2016; Liu et al., 2017). The yield of pakchoi was found<38.4 t/ha and plant critical N content was less than 4.78%; then, N supply was found essential for pakchoi growth and development. Similar responses to the NNI have been reported for corn and wheat (Ziadi et al., 2008; Ziadi et al., 2010), and the NNI is used as a reference method for detecting N deficiency. In addition, the relationship between the NNI and N_c_ can be used to determine the requirement of further N fertilization directly in plants. The remote sensing technology has been reported in using the NNI and N_c_ as a diagnostic tool for determining the crop biomass and N uptake in plants (Wei et al., 2012). Non-destructive simulation based models are used to construct the N dilution curve using the NNI and N_c_ in tomatoes (Jing et al., 2020) and other crops like cucumber, zucchini, melon, and pepper (Gallardo et al., 2016) for the N demand status with the coefficient of determination (R^2^) values ranging from 92%–99% under different N applications.

In this study, the results showed an increasing trend in N contents in pakchoi and then declined after a certain N level and growing stage. The reason for N dilution in plants is that as they expand their canopy area, the plants’ lower parts cannot absorb light properly. Younger leaves cannot fully expand due to light shortages, leading to a decrease in leaf N content. The N uptake and photosynthetic rate per unit leaf area are higher for older leaves at the canopy top. The N elements present in older leaves are used for producing new leaves, which block the other leaves and decrease the N contents in plants (Prioul et al., 1980; Bahmani et al., 2000; Hikosaka, 2005; Liu et al., 2017). A decrease in N content in plants due to an imbalance in photothermal effects leads to the vertical distribution of N and a reduction in plant biomass (Trouwborst et al., 2010; Chen et al., 2018). There is no competition for light among plant growth and decline in the N_c_ content with biomass accumulation (Liu et al., 2017). Similar results were found in present studies; when LFT increased, FW, DW, and N accumulation increased in pakchoi under different N applications but decreased with the increase of N application after a certain level.

### Nitrogen supply model of pakchoi based on multiparameter fusion method

The precise N supply in crops requires accurate N monitoring and demand prediction. Non-destructive remote sensing technology in agriculture is an emerging field for N diagnosis in plants (Xiong et al., 2019; Chang et al., 2021). Combining remote sensing technology with the N demand model to determine crop N requirement accurately is a new idea for determining the accurate N management in crops (Talaviya et al., 2020; Subeesh and Mehta, 2021). A critical N content model of pakchoi through analyzing the relationship between aboveground N content and biomass based on phenotypic image acquisition and machine learning technology to develop a relationship between the crop phenotype of non-heading cabbage and the N demand status in pakchoi (Xiong et al., 2019). A model based on N supply was constructed to develop a relationship between aboveground N content and biomass along with visible light images in pakchoi for the N prediction model. In the present study, the phenotypic features were used as input parameters. N demand models were developed to evaluate the NNI of pakchoi (**Supplementary Tables 2, 3**). The *R^2^* values of the three models reached approximately 0.90, and the *RMSE* value was found lower than 0.1. The results revealed that phenotypic imaging combined with a machine learning algorithm could effectively evaluate plants’ NNI status. We assessed the NNI prediction models throughout the entire growth cycle of pakchoi. The precise prediction of the NNI at the seedling stage indicated that the prediction models can be used to guide N topdressing. The precise prediction of the NNI at the harvest stage is found beneficial for assessing the N uptake and calculating the N consumption by the pakchoi (Xiong et al., 2019). The machine learning models exhibited outstanding robustness and applicability under different scenarios and thus can be used in practice for the N demand status in pakchoi (Xiong et al., 2019). Different studies using machine learning techniques have been reported to determine the N demand status in rice (Islam et al., 2020; Iatrou et al., 2021). We also explored the effects of the photo effect and temperature on yield. Multiple factors such as phenotype, meteorology, and management practices were fused to construct the N demand model of pakchoi while considering N use efficiency and biomass to predict the model.

The N demand on N_c_ and machine learning techniques was based on the actual crop growth, with the advantages over other developed models: crop specific, precise, simple, and biologically sound. These diagnostic tools based on the Nc dilution curve for pakchoi can be utilized directly to assess the crop nutrition status and recommend further fertilization during different growing stages. However, further field studies are still required to validate NNI usage as a diagnostic tool with N fertilization for other regions under different growing and climatic conditions. The present study proposed an N supply model and designed an N supply decision-making framework system based on the critical N_c_ curve and phenotype characteristics.

## Conclusion

In conclusion, the N_c_ dilution curve based on plant biomass for pakchoi were developed ( N_*c*_%=4.78×*DW* ^−0.33^ .)The results of this study revealed that as the DW of pakchoi was not higher than 1.5 t/ha, the critical N_c_ of the aboveground part was 4.78%, a constant value. However, when DW was over 1.5 t/ha, the critical N content began to decline. The yield of pakchoi was less than 38.4 t/ha and plant-critical N content was less than 4.78%. Then, N supply was found essential for pakchoi growth. We also established an N demand model based on the critical N content, phenotype, and biomass accumulation of pakchoi. Furthermore, we verified the model with separate experimental data. The results revealed that the prediction and actual value of crop N demand was above 90% and values were perfectly fitted to the model values (R^2^ = 0.948, RMSE = 1.96 mg plant^-1^). This study can provide a theoretical and technical basis for precise N management in pakchoi and other vegetable production.

## Data availability statement

The original contributions presented in the study are included in the article/**Supplementary Material**. Further inquiries can be directed to the corresponding author.

## Author contributions

LY and QN designed research. XX performed research and analyzed data. XX, MH and DH wrote and revised the paper. All authors contributed to the article and approved the submitted version.

## References

[B1] Abdallah F. B. Olivier M. Goffart J. P. Minet O. (2016). Establishing the nitrogen dilution curve for potato cultivar bintje in Belgium. Potato Res. 59 (3), 241–258. doi: 10.1007/s11540-016-9331-y

[B2] Anas M. Liao F. Verma K. K. Sarwar M. A. Mahmood A. Chen Z. L.. (2020). Fate of nitrogen in agriculture and environment: Agronomic, eco-physiological and molecular approaches to improve nitrogen use efficiency. Biol. Res. 53 (1), 1–20. doi: 10.1186/s40659-020-00312-4 33066819 PMC7565752

[B3] Bahmani I. Hazard L. Varlet-Grancher C. Betin M. Lemaire G. Matthew C.. (2000). Differences in tillering of long-and short-leaved perennial ryegrass genetic lines under full light and shade treatments. Crop Sci. 40 (4), 1095–1102. doi: 10.2135/cropsci2000.4041095x

[B4] Bassirirad H. (2000). Kinetics of nutrient uptake by roots: responses to global change. New Phytol. 147 (1), 155–169. doi: 10.1046/j.1469-8137.2000.00682.x

[B5] Bo Y. He H. B. Xu H. C. Zhu T. Z. Tao L. Jian K.. (2021). Determining nitrogen status and quantifying nitrogen fertilizer requirement using a critical nitrogen dilution curve for hybrid indica rice under mechanical pot-seedling transplanting pattern. J. Integr. Agric. 20 (6), 1474–1486. doi: 10.1016/S2095-3119(21)63622-5

[B6] Chang L. Li D. Hameed M. K. Yin Y. Huang D. Niu Q. (2021). Using a hybrid neural network model DCNN–LSTM for image-based nitrogen nutrition diagnosis in muskmelon. Horticulturae. 7 (11), 489. doi: 10.3390/horticulturae7110489

[B7] Chen J. B. Dong C. C. Yao X. D. Wang W. (2018). Effects of nitrogen addition on plant biomass and tissue elemental content in different degradation stages of temperate steppe in northern China. J. Plant Ecol. 11 (5), 730–739. doi: 10.1093/jpe/rtx035

[B8] Ciampitti I. van Versendaal E. Rybecky J. F. Lacasa J. Fernandez J. Makowski D.. (2022). A global dataset to parametrize critical nitrogen dilution curves for major crop species. Sci. Data 9 (1), 1–11. doi: 10.1038/s41597-022-01395-2 35672371 PMC9174182

[B9] de Paz J. M. Ramos C. Visconti F. (2022). Critical nitrogen dilution curve and dry matter production parameters for several Mediterranean vegetables. Sci. Hortic. 303, 111194. doi: 10.1016/j.scienta.2022.111194

[B10] Du L. Li Q. Li L. Wu Y. Zhou F. Liu B.. (2020). Construction of a critical nitrogen dilution curve for maize in southwest China. Sci. Rep. 10 (1), 1–10. doi: 10.1038/s41598-020-70065-3 32753694 PMC7403409

[B11] Durand J. L. Gonzalez-Dugo V. Gastal F. (2010). How much do water deficits alter the nitrogen nutrition status of forage crops? Nutr. Cycling Agroecosyst. 88 (2), 231–243. doi: 10.1007/s10705-009-9330-3

[B12] Ekbladh G. Witter E. (2010). Determination of the critical nitrogen concentration of white cabbage. Eur. J. Agron. 33 (4), 276–284. doi: 10.1016/j.eja.2010.08.001

[B13] Errecart P. M. Agnusdei M. G. Lattanzi F. A. Marino M. A. Berone G. D. (2014). Critical nitrogen concentration declines with soil water availability in tall fescue. Crop Sci. 54 (1), 318–330. doi: 10.2135/cropsci2013.08.0561

[B14] Fahey J. W. (2015). Brassica: Characteristics and properties. Encyclopedia Food Health, 469–477. doi: 10.1016/B978-0-12-384947-2.00083-0

[B15] FAOSTAT (2022) Food and agriculture organization of the united nations. Available at: https://www.fao.org/faostat.

[B16] Fatemi H. Zaghdoud C. Nortes P. A. Carvajal M. Martínez-Ballesta M. D. C. (2020). Differential aquaporin response to distinct effects of two zn concentrations after foliar application in pak choi (*Brassica rapa l*.) plants. Agronomy 10 (3), 450. doi: 10.3390/agronomy10030450

[B17] Fogaça M. A. D. F. Andriolo J. L. Godoi R. D. S. Barros C. A. P. D. Janisch D. I. Vaz M. A. B. (2008). Nitrogen critical dilution curve for the muskmelon crop. Ciec. Rural. 38, 345–350. doi: 10.1590/S0103-84782008000200008

[B18] Foulkes M. J. Sylvester-Bradley R. Scott R. K. (1998). Evidence for differences between winter wheat cultivars in acquisition of soil mineral nitrogen and uptake and utilization of applied fertilizer nitrogen. J. Agric. Sci. 130 (1), 29–44. doi: 10.1017/S0021859697005029

[B19] Frede K. Schreiner M. Zrenner R. Graefe J. Baldermann S. (2018). Carotenoid biosynthesis of pak choi (*Brassica rapa ssp. chinensis*) sprouts grown under different light-emitting diodes during the diurnal course. Photochem. Photobiol. Sci. 17 (10), 1289–1300. doi: 10.1039/c8pp00136g 30065986

[B20] Gallardo M. Fernández M. D. Giménez C. Padilla F. M. Thompson R. B. (2016). Revised VegSyst model to calculate dry matter production, critical n uptake and ETc of several vegetable species grown in Mediterranean greenhouses. Agric. Syst. 146, 30–43. doi: 10.1016/j.agsy.2016.03.014

[B21] Greenwood D. J. Lemaire G. Gosse G. Cruz P. Draycott A. Neeteson J. J. (1990). Decline in percentage n of C3 and C4 crops with increasing plant mass. Ann. Bot. 66 (4), 425–436. doi: 10.1093/oxfordjournals.aob.a088044

[B22] Gupta S. Gowri B. S. Lakshmi A. J. Prakash J. (2013). Retention of nutrients in green leafy vegetables on dehydration. J. Food Sci. Technol. 50 (5), 918–925. doi: 10.1007/s13197-011-0407-z 24425998 PMC3722389

[B23] Haegele J. W. Cook K. A. Nichols D. M. Below F. E. (2013). Changes in nitrogen use traits associated with genetic improvement for grain yield of maize hybrids released in different decades. Crop Sci. 53 (4), 1256–1268. doi: 10.2135/cropsci2012.07.0429

[B24] Hameed M. K. Umar W. Razzaq A. Aziz T. Maqsood M. A. Wei S.. (2022). Differential metabolic responses of lettuce grown in soil, substrate and hydroponic cultivation systems under NH_4_ ^+^/NO_3_ ^–^ application. Metabolites 12 (5), 444. doi: 10.3390/metabo12050444 35629948 PMC9143640

[B25] Hanson P. Yang R. Y. Chang L. C. Ledesma L. Ledesma D. (2009). Contents of carotenoids, ascorbic acid, minerals and total glucosinolates in leafy brassica pakchoi (*Brassica rapa l. chinensis*) as affected by season and variety. J. Sci. Food Agric. 89 (5), 906–914. doi: 10.1002/jsfa.3533

[B26] He Z. Qiu X. Ata-Ul-Karim S. T. Li Y. Liu X. Cao Q.. (2017). Development of a critical nitrogen dilution curve of double cropping rice in south China. Front. Plant Sci. 8, 638. doi: 10.3389/fpls.2017.00638 28503181 PMC5408224

[B27] Hikosaka K. (2005). Leaf canopy as a dynamic system: ecophysiology and optimality in leaf turnover. Ann. Bot. 95 (3), 521–533. doi: 10.1093/aob/mci050 15585542 PMC4246797

[B28] Iatrou M. Karydas C. Iatrou G. Pitsiorlas I. Aschonitis V. Raptis I.. (2021). Topdressing nitrogen demand prediction in rice crop using machine learning systems. Agriculture. 11 (4), 312. doi: 10.3390/agriculture11040312

[B29] Islam T. Rizan R. U. B. Tusher Y. A. Shafiuzzaman M. Hossain M. A. Galib S. (2020). Nitrogen fertilizer recommendation for paddies through automating the leaf color chart (LCC). Intl. J. Adv. Comput. Sci. Appl. 11 (8). doi: 10.14569/IJACSA.2020.0110891

[B30] Jing B. Niu N. Zhang W. Wang J. Diao M. (2020). 15N tracer-based analysis of fertiliser nitrogen accumulation, utilisation and distribution in processing tomato at different growth stages. Acta Agric. Scand. Sect B. 70 (8), 620–627. doi: 10.1080/09064710.2020.1825786

[B31] Justes E. Jeuffroy M. H. Mary B. (1997). “Wheat, barley, and durum wheat,” in Diagnosis of the nitrogen status in crops (Berlin, Heidelberg: Springer), 73–91.

[B32] Justes E. Mary B. Meynard J. M. Machet J. M. Thelier-Huché L. (1994). Determination of a critical nitrogen dilution curve for winter wheat crops. Ann. Bot. 74 (4), 397–407. doi: 10.1006/anbo.1994.1133

[B33] Lemaire G. Jeuffroy M. H. Gastal F. (2008). Diagnosis tool for plant and crop n status in vegetative stage: Theory and practices for crop n management. Eur. J. Agron. 28 (4), 614–624. doi: 10.1016/j.eja.2008.01.005

[B34] Lemaire G. Salette J. (1984). Relationship between growth and nitrogen uptake in a pure grass stand. I.-Environmental effects. doi: 10.1016/j.fcr.2006.05.009

[B35] Lemaire G. van Oosterom E. Sheehy J. Jeuffroy M. H. Massignam A. Rossato L. (2007). Is crop n demand more closely related to dry matter accumulation or leaf area expansion during vegetative growth? Field Crops Res. 100 (1), 91–106. doi: 10.22069/ijpp.2017.3546

[B36] Li S. Bañuelos G. S. Min J. Shi W. (2015). Effect of continuous application of inorganic nitrogen fertilizer on selenium concentration in vegetables grown in the taihu lake region of China. Plant Soil. 393 (1), 351–360. doi: 10.1007/s11104-015-2496-3

[B37] Liu B. Asseng S. Wang A. Wang S. Tang L. Cao W.. (2017). Modelling the effects of post-heading heat stress on biomass growth of winter wheat. Agric. For. Meteorol. 247, 476–490. doi: 10.1016/j.agrformet.2017.08.018

[B38] Liu B. Liu L. Asseng S. Zou X. Li J. Cao W.. (2016). Modelling the effects of heat stress on post-heading durations in wheat: A comparison of temperature response routines. Agric. For. Meteorol. 222, 45–58. doi: 10.1016/j.agrformet.2016.03.006

[B39] Min J. Zhang H. Shi W. (2012). Optimizing nitrogen input to reduce nitrate leaching loss in greenhouse vegetable production. Agric. Water Manage. 111, 53–59. doi: 10.1016/j.agwat.2012.05.003

[B40] Nunn C. Hastings A. F. S. J. Kalinina O. Özgüven M. Schüle H. Tarakanov I. G.. (2017). Environmental influences on the growing season duration and ripening of diverse miscanthus germplasm grown in six countries. Front. Plant Sci. 8, 907. doi: 10.3389/fpls.2017.00907 28611816 PMC5447773

[B41] Prioul J. L. Brangeon J. Reyss A. (1980). Interaction between external and internal conditions in the development of photosynthetic features in a grass leaf: II. reversibility of light-induced responses as a function of developmental stages. Plant Physiol. 66 (4), 770–774. doi: 10.1104/pp.66.4.770 16661519 PMC440720

[B42] Qiang S. C. Zhang F. C. Dyck M. Zhang Y. Xiang Y. Z. Fan J. L. (2019). Determination of critical nitrogen dilution curve based on leaf area index for winter wheat in the guanzhong plain, Northwest China. J. Integr. Agric. 18 (10), 2369–2380. doi: 10.1016/S2095-3119(19)62688-2

[B43] Reis S. Bekunda M. Howard C. M. Karanja N. Winiwarter W. Yan X.. (2016). Synthesis and review: tackling the nitrogen management challenge: from global to local scales. Environ. Res. Lett. 11 (12), 120205. doi: 10.1088/1748-9326/11/12/120205

[B44] Sadras V. O. Lawson C. (2013). Nitrogen and water-use efficiency of Australian wheat varieties released between 1958 and 2007. Eur. J. Agron. 46, 34–41. doi: 10.1016/j.eja.2012.11.008

[B45] Saki T. Yomi M. Rajashekhar Rao B. K. (2019). Critical nitrogen content and nitrogen nutrition index for sweetpotato crop. J. Plant Nutr. 42 (15), 1750–1759. doi: 10.1080/01904167.2019.1648685

[B46] Sassenrath G. F. Schneider J. M. Gaj R. Grzebisz W. Halloran J. M. (2013). Nitrogen balance as an indicator of environmental impact: Toward sustainable agricultural production. Renewable Agric. Food Syst. 28 (3), 276–289. doi: 10.1017/S1742170512000166

[B47] Sedlář O. Balík J. Černý J. Kulhánek M. Vašák F. (2017). Relation between nitrogen nutrition index and production of spring malting barley. Int. J. Plant Prod. 11 (3), 379–388. doi: 10.1016/S1002-0160(13)60082-X

[B48] Shan-Chao Y. U. E. Fu-Lai S. U. N. Qing-Feng M. E. N. G. Rong-Fang Z. H. A. O. Fei L. I. Xin-Ping C. H. E. N.. (2014). Validation of a critical nitrogen curve for summer maize in the north China plain. Pedosphere 24 (1), 76–83. doi: 10.1016/S2095-3119(12)60457-2

[B49] Shlevin E. Zilberman A. Ben-Asher J. (2018). Theoretical determination of a critical nitrogen dilution curve based on the carrot case study. Agric. Res. 7 (2), 239–244. doi: 10.1007/s40003-018-0303-0

[B50] Sinclair T. R. Rufty T. W. (2012). Nitrogen and water resources commonly limit crop yield increases, not necessarily plant genetics. Global Food Secur. 1 (2), 94–98. doi: 10.1016/j.gfs.2012.07.001

[B51] Stockle C. O. Debaeke P. (1997). “Modeling crop n requirement: A critical analysis,” in Proc. 4th European society of agronomy congress(Wageningen).

[B52] Stöckle C. O. Donatelli M. Nelson R. (2003). CropSyst, a cropping systems simulation model. Eur. J. Agron. 18 (3-4), 289–307. doi: 10.1016/S1161-0301(02)00109-0

[B53] Stockle C. O. Martin S. A. Campbell G. S. (1994). CropSyst, a cropping systems simulation model: water/nitrogen budgets and crop yield. Agric. Syst. 46 (3), 335–359. doi: 10.1016/0308-521X(94)90006-2

[B54] Stockle C. O. Nelson R. L. (1996). Cropsyst user’s manual (Version 2.0) biological systems engineering department (Pullman, WA, USA: Washington State University).

[B55] Subeesh A. Mehta C. R. (2021). Automation and digitization of agriculture using artificial intelligence and internet of things. Artif. Intell. Agric. 5, 278–291. doi: 10.1016/j.aiia.2021.11.004

[B56] Sun J. Li W. Li C. Chang W. Zhang S. Zeng Y.. (2020). Effect of different rates of nitrogen fertilization on crop yield, soil properties and leaf physiological attributes in banana under subtropical regions of China. Front. Plant Sci. 11, 613760. doi: 10.3389/fpls.2020.613760 33408734 PMC7779679

[B57] Talaviya T. Shah D. Patel N. Yagnik H. Shah M. (2020). Implementation of artificial intelligence in agriculture for optimisation of irrigation and application of pesticides and herbicides. Artif. Intell. Agric. 4, 58–73. doi: 10.1016/j.aiia.2020.04.002

[B58] Tei F. De Neve S. de Haan J. Kristensen H. L. (2020). Nitrogen management of vegetable crops. Agric. Water Manage. 240, 106316. doi: 10.1016/j.agwat.2020.106316

[B59] Trouwborst G. Oosterkamp J. Hogewoning S. W. Harbinson J. Van Ieperen W. (2010). The responses of light interception, photosynthesis and fruit yield of cucumber to LED-lighting within the canopy. Physiol. Plant 138 (3), 289–300. doi: 10.1111/j.1399-3054.2009.01333.x 20051030

[B60] Valenciana G. (2017). Informe para la modificación de la ley 10/2010, de la generalitat, de ordenación y gestión de la función pública valenciana. Monogràfics Drets 2, 5–231. doi: 10.3390/agronomy10091257

[B61] Wang X. Ma F. Diao M. Fan H. Cui J. Jia B.. (2013). Simulation of critical nitrogen concentration, nitrogen uptake and nitrogen nutrition index of processing tomato with drip irrigation. Trans. Chin. Soc Agric. Eng. 29 (18), 99–108.

[B62] Wei W. Xia Y. Tian Y. C. Liu X. J. Jun N. I. Cao W. X.. (2012). Common spectral bands and optimum vegetation indices for monitoring leaf nitrogen accumulation in rice and wheat. J. Integr. Agric. 11 (12), 2001–2012.

[B63] Wei W. Yang M. Liu Y. Huang H. Ye C. Zheng J.. (2018). Fertilizer n application rate impacts plant-soil feedback in a sanqi production system. Sci. Total Environ. 633, 796–807. doi: 10.1016/j.scitotenv.2018.03.219 29602118

[B64] Xiaochuang C. Lianghuan W. Ling Y. Xiaoyan L. Yuanhong Z. Qianyu J. (2015). Uptake and uptake kinetics of nitrate, ammonium and glycine by pakchoi seedlings (Brassica campestris l. ssp. chinensis l. makino). Sci. Hortic. 186, 247–253. doi: 10.1016/j.scienta.2015.02.010

[B65] Xie Y. Wang S. Luo C. Sun M. Wang Y. Yang J.. (2020). Using plastic mulching improves greenhouse-grown pakchoi (Brassica rapa subsp. chinensis) growth and water use efficiency under irrigation scheduling based on soil water content. Agronomy 10 (9), 1257.

[B66] Xiong X. Zhang J. Guo D. Chang L. Huang D. (2019). Non-invasive sensing of nitrogen in plant using digital images and machine learning for brassica campestris ssp. chinensis l. Sensors. 19 (11), 2448. doi: 10.3390/s19112448 31146350 PMC6603544

[B67] Yan X. Ti C. Vitousek P. Chen D. Leip A. Cai Z.. (2014). Fertilizer nitrogen recovery efficiencies in crop production systems of China with and without consideration of the residual effect of nitrogen. Environ. Res. Lett. 9 (9), 095002. doi: 10.1088/1748-9326/9/9/095002

[B68] Ye C. Liu Y. Liu J. Li Y. Sun B. Shu S.. (2021). Simulation of the critical nitrogen dilution curve in Jiangxi double-cropped rice region based on leaf dry matter weight. PloS One 16 (11), e0259204. doi: 10.1371/journal.pone.0259204 34731196 PMC8565756

[B69] Yin M. Li Y. Xu L. Shen S. Fang H. (2018). Nutrition diagnosis for nitrogen in winter wheat based on critical nitrogen dilution curves. Crop Sci. 58 (1), 416–425. doi: 10.2135/cropsci2017.05.0326

[B70] Yousaf M. Li J. Lu J. Ren T. Cong R. Fahad S.. (2017). Effects of fertilization on crop production and nutrient-supplying capacity under rice-oilseed rape rotation system. Sci. Rep. 7 (1), 1–9. doi: 10.1038/s41598-017-01412-0 28455510 PMC5430767

[B71] Yu F. H. Bai J. C. Jin Z. Y. Guo Z. H. Yang J. X. Chen C. L. (2022). Combining the critical nitrogen concentration and machine learning algorithms to estimate nitrogen deficiency in rice from UAV hyperspectral data. J. Integr. Agric. doi: 10.1016/j.jia.2022.12.007

[B72] Zhang M. Yao Y. Tian Y. Ceng K. Zhao M. Zhao M.. (2018). Increasing yield and n use efficiency with organic fertilizer in Chinese intensive rice cropping systems. Field Crops Res. 227, 102–109. doi: 10.1016/j.fcr.2018.08.010

[B73] Zhao B. Ata-UI-Karim S. T. Yao X. Tian Y. Cao W. Zhu Y.. (2016). A new curve of critical nitrogen concentration based on spike dry matter for winter wheat in eastern China. PloS One 11 (10), e0164545. doi: 10.1371/journal.pone.0164545 27732634 PMC5061393

[B74] Zhao B. Ata-Ul-Karim S. T. Liu Z. Ning D. Xiao J. Liu Z.. (2017). Development of a critical nitrogen dilution curve based on leaf dry matter for summer maize. Field Crops Res. 208, 60–68. doi: 10.1016/j.fcr.2017.03.010

[B75] Zhao C. Liu G. Chen Y. Jiang Y. Shi Y. Zhao L.. (2022). Excessive nitrogen application leads to lower rice yield and grain quality by inhibiting the grain filling of inferior grains. Agriculture 12 (7), 962. doi: 10.3390/agriculture12070962

[B76] Zhao C. Liu L. Wang J. Huang W. Song X. Li C. (2005). Predicting grain protein content of winter wheat using remote sensing data based on nitrogen status and water stress. Int. J. Appl. Earth Obs. Geoinf. 7 (1), 1–9. doi: 10.1016/j.jag.2004.10.002

[B77] Zhou T. M. Zhen W. U. Wang Y. C. Su X. J. Qin C. X. Huo H. Q.. (2019). Modelling seedling development using thermal effectiveness and photosynthetically active radiation. J. Integr. Agric. 18 (11), 2521–2533. doi: 10.1016/S2095-3119(19)62671-7

[B78] Zhu B. Yang J. Zhu Z. J. (2013). Variation in glucosinolates in pakchoi cultivars and various organs at different stages of vegetative growth during the harvest period. J. Zhejiang Uni. Sci. B. 14 (4), 309–317. doi: 10.1631/jzus.B1200213 PMC3625527 23549848

[B79] Ziadi N. Bélanger G. Claessens A. Lefebvre L. Cambouris A. N. Tremblay N.. (2010). Determination of a critical nitrogen dilution curve for spring wheat. Agron. J. 102 (1), 241–250. doi: 10.2134/agronj2009.0266

[B80] Ziadi N. Brassard M. Bélanger G. Cambouris A. N. Tremblay N. Nolin M. C.. (2008). Critical nitrogen curve and nitrogen nutrition index for corn in eastern Canada. Agron. J. 100 (2), 271–276. doi: 10.2134/agronj2007.0059

