# Supplementary material for: Study on nitrogen demand model in pakchoi (Brassica campestris ssp. Chinensis L.) based on nitrogen contents and phenotypic characteristics
Source: Front Plant Sci. 2023 Feb 15;14:1111216. doi: 10.3389/fpls.2023.1111216 (PMC9975592; doi:10.3389/fpls.2023.1111216)
Supplement: Supplementary file 1 [file DataSheet_1.docx]

**
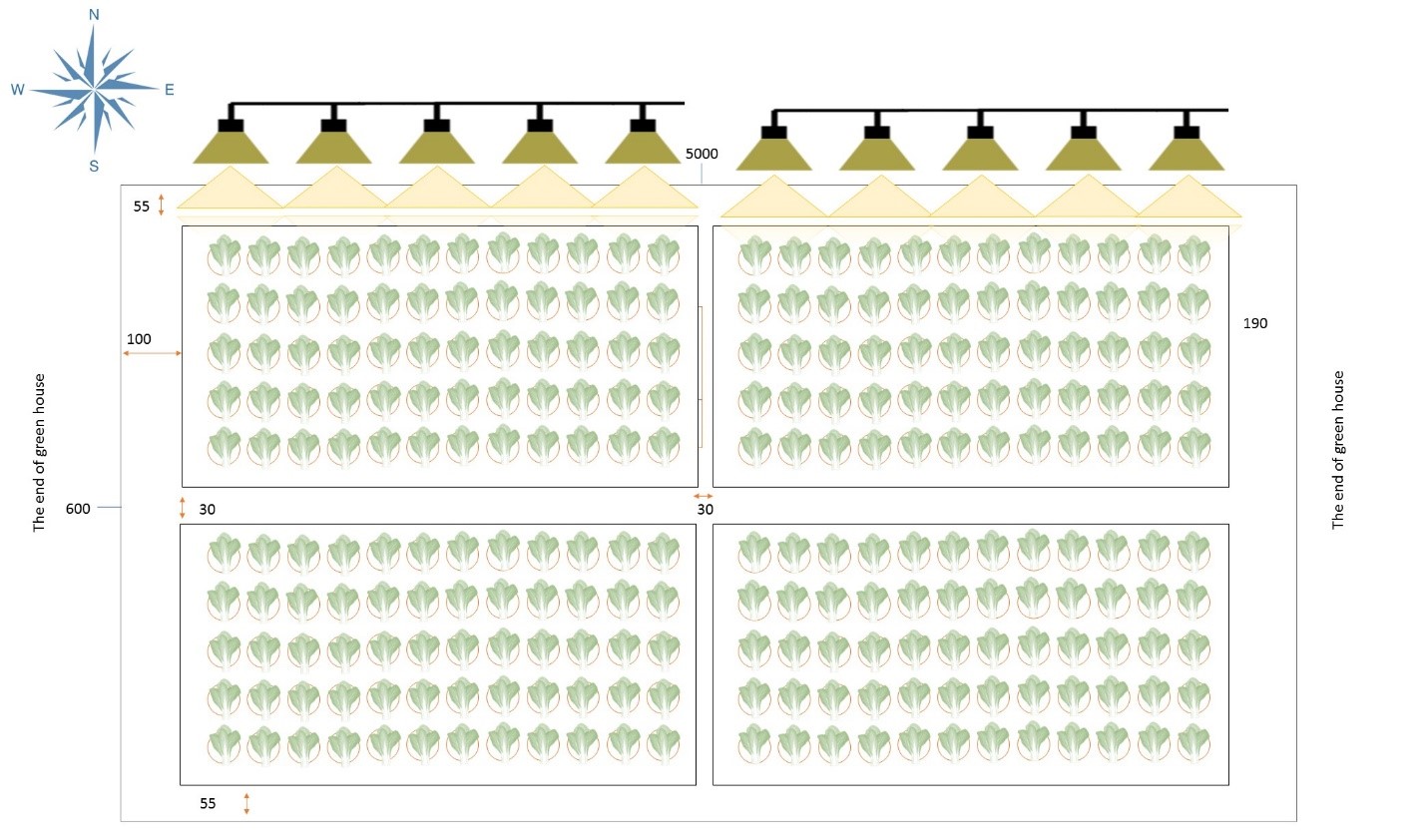
 Supplementary figure S1** ǀ An overview of greenhouse pakchoi growing experiments. Random plant samples collected from experiments. The units of numbers in the figure are centimeters (cm).

**Photothermal effect calculation**

The [photosynthetically](file:///E:\%E6%9C%89%E9%81%93\Dict\7.5.2.0\resultui\dict\?keyword=photosynthetically)[active](file:///E:\%E6%9C%89%E9%81%93\Dict\7.5.2.0\resultui\dict\?keyword=active)[radiation](file:///E:\%E6%9C%89%E9%81%93\Dict\7.5.2.0\resultui\dict\?keyword=radiation) (PAR) and temperature (T) are the most critical environmental factors affecting crop growth [1]. Both light effect (*f*_I_) and temperature thermal effects (*f*_T_) affect biomass and nutrient accumulation in plants. The product of *f*_I_ and *f*_T_ is defined as a photothermal effect (LTF). T and PAR data during plant growth are used to establish photothermal effect and nutrition dynamic relationship of plant biomass to predict the full expansion of the first true leaf in plants during the growth days [2]. However, we used LTF to evaluate the effect of temperature and radiation on yield, DW accumulation, and N content in aboveground parts of pakchoi.

**N content model**

The cropping systems simulation (CropSyst) model is based on N_c_ [3] and employs the indexes of maximum N contents (N_max_), critical N contents (N_c_), and a minimum of N contents (N_min_) in the N content curve. The N content in plants is higher than N_c_, and the plant is not subjected to N stress, but the maximum N absorption amount is less than N_max_. However, the plant’s N content is lower than N_c_, and the plant’s growth is inhibited. Additionally, the plant N content is lower than N_min_, and plants are not grown anymore [4].

**Actual N content (N_a,_ %)**

Following phenotypic characteristics were extracted in all experiments as described by Xiong et al. [5] and shown in **supplementary** **table 3**

1) Input 23 phenotypic parameters;

2) Output N content (N_a_, %);

3) Random forest algorithm, *n*_tree_=300, mtry=2

**
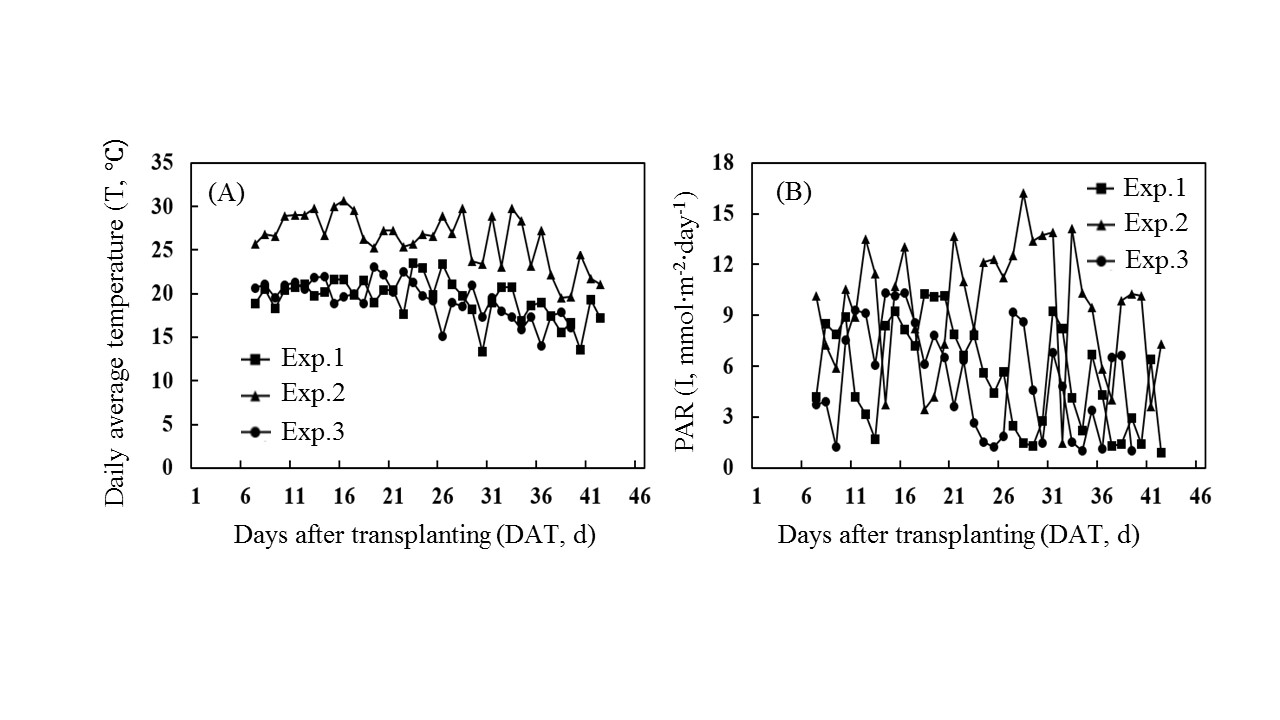
Supplementary figure S2ǀ** The daily average (A) temperature and (B) [photosynthetically](file:///E:\%E6%9C%89%E9%81%93\Dict\7.5.2.0\resultui\dict\?keyword=photosynthetically) [active](file:///E:\%E6%9C%89%E9%81%93\Dict\7.5.2.0\resultui\dict\?keyword=active) [radiation](file:///E:\%E6%9C%89%E9%81%93\Dict\7.5.2.0\resultui\dict\?keyword=radiation) for the whole plant growth cycle in three experiments


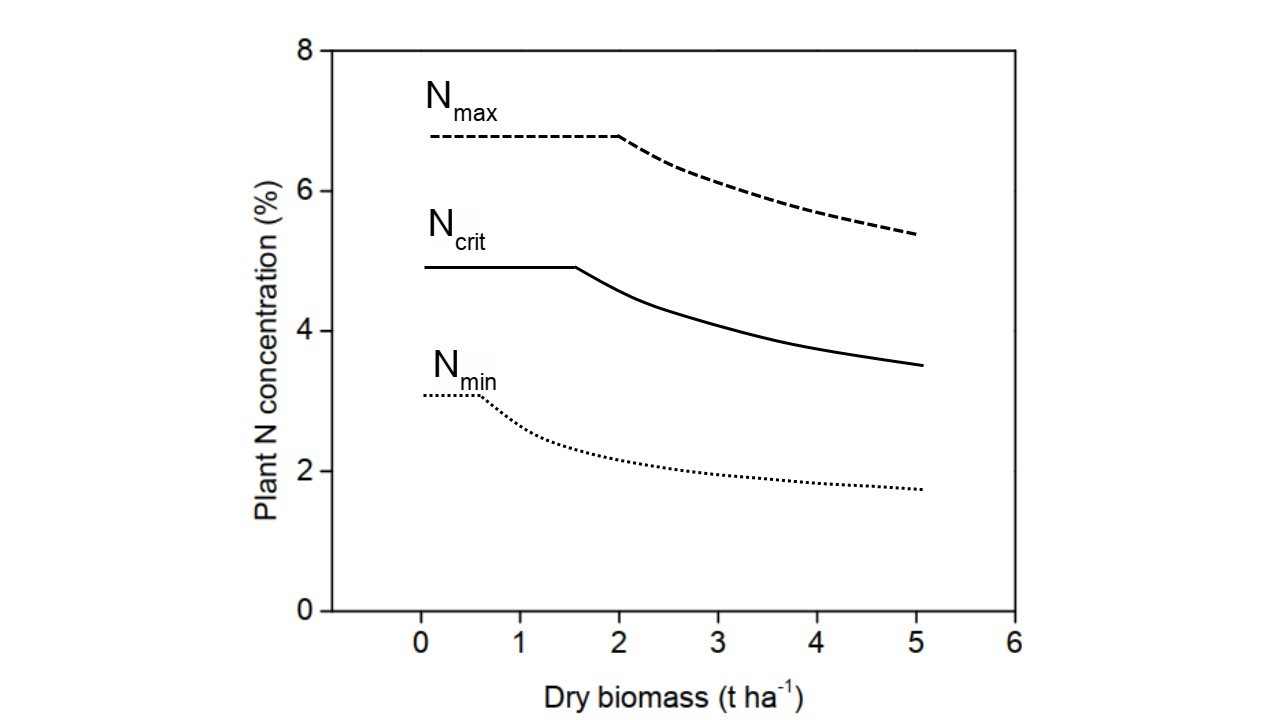
 **Supplementary figure S3** ǀ Nitrogen concentration curve pattern with dry biomass with minimum, critical and maximum nitrogen concentrations in pakchoi

Note: N_max_ represents the maximum nitrogen concentration curve, N_crit_ represents the critical nitrogen concentration curve, and N_min_ represents the minimum nitrogen concentration curve.

**Supplementary table 1** **ǀ** Meteorological conditions and growth days of three experiments

| Experiment No. | Avg T（℃） | Accum T (℃) | | SR (mol/m^2^） | Accum PR (mol/m^2^） | Growth days (DAT, d) |
| --- | --- | --- | --- | --- | --- | --- |
| Exp 1 | 19.4 | 698.4 | 5.5 | | 197.3 | 49 |
| Exp 2 | 26.3 | 945.1 | 9.5 | | 343.1 | 42 |
| Exp 3 | 19.3 | 637.4 | 5.3 | | 174.7 | 42 |

**Note:** Abbreviation represents Avg T= The average daily temperature; Accum T= Accumulated temperature; SR= average effective sunlight radiation; Acc PR= Accumulated photosynthetically effective radiation; DAT= Days after transplanting.

**Model Evaluation under Different Plant N Nutrition Status**

To check whether our models can be generalized across different scenarios, we applied the models under N surplus and deficiency conditions to predict the NNI. The *R^2^*, *RE*, and accuracy were selected as the evaluation indicators. Supplementary table 2 lists the evaluation results. Under the N surplus condition, the *R^2^* and accuracy of the three models were very low; however, the RE was very low. The models’ performance in predicting excessive N in the pakchoi was poor. Under N deficiency, the *R^2^* of the three models crossed 0.90, and the accuracy reached approximately 0.95. However, the RE value of only the RF model was lower than 10%, indicating that the performance of this model was the best.

The color, texture, and morphological-related features were used as input parameters. RF, SVR, and NN models were developed to evaluate the NNI of pakchoi. The *R^2^* values of the three models reached approximately 0.900, and the RMSE value was lower than 0.1. The results revealed that phenotypic imaging combined with a machine learning algorithm could effectively evaluate the plants’ nitrogen nutrition status.

**Supplementary table 2 ǀ** Model evaluation result under different plant N nutrition status and growth stages

| Different Scenarios | Range of Measure NNI | Model | Model Evaluation Results | | | |
| --- | --- | --- | --- | --- | --- | --- |
|  |  |  | Range of Simulated NNI | R^2^ | Relative Error (%) | Accuracy |
| Excessive | 1.01~1.12 | RF | 0.895~1.101 | 0.470 | 2.57 | 0.787 |
|  |  | SVR | 0.772~1.135 | 0.206 | 3.56 | 0.586 |
|  |  | NN | 0.822~1.000 | 0.016 | 1.76 | 0.085 |
| Low | 0.26~0.93 | RF | 0.262~1.081 | 0.945 | 8.35 | 0.948 |
|  |  | SVR | 0.173~1.087 | 0.921 | 10.51 | 0.984 |
|  |  | NN | 0.237~1.000 | 0.918 | 10.35 | 0.952 |
| Seedling period | 0.63~1.08 | RF | 0.495~1.068 | 0.795 | 6.56 | 0.823 |
|  |  | SVR | 0.348~1.084 | 0.703 | 8.79 | 0.856 |
|  |  | NN | 0.416~1.000 | 0.674 | 8.35 | 0.766 |
| Harvest period | 0.26~1.12 | RF | 0.262~1.101 | 0.985 | 4.49 | 0.943 |
|  |  | SVR | 0.173~1.135 | 0.981 | 5.23 | 0.974 |
|  |  | NN | 0.237~1.000 | 0.969 | 6.06 | 0.869 |

Note: RF, Random Forest; SVR, Support Vector Regression; NN, Neural Network

**Supplementary table 3** **ǀ** Phenotypic traits used for model development

| Category | Trait name | Description |
| --- | --- | --- |
|  |  |  |
| Color | R_mean | Average color in the red range of the RGB color space |
|  | R_median | The median of R in the RGB color space |
|  | G_mean | Average color in the green range of the RGB color space |
|  | G_median | The median of G in the RGB color space |
|  | B_mean | Average color in the blue range of the RGB color space |
|  | B_median | The median of B in the RGB color space |
|  | l_mean | The mean of L channel in LAB color space |
|  | l_median | The median of L channel in LAB color space |
|  | b_mean | The mean of B channel in LAB color space |
|  | b_median | The median of B channel in LAB color space |
|  | H_mean | Average hue in the HSV color space |
|  | H_median | The median of H in the HSV color space |
|  | S_mean | Average saturation in HSV color space |
|  | S_median | The median of S in the HSV color space |
|  | V_mean | Average value in HSV color space |
|  | V_median | The median of V in the HSV color space |
| Texture | homogeneity | The local changes of image texture |
|  | energy | The degree of thickness and uniformity for texture |
|  | ASM | Angular second moment |
| Morphology | contour_area | Area of plant contour |
|  | hull_area | Convex hull area (mm^2^) |
|  | r | The radius of the minimum circumscribed circle |
|  | equivalent_diameter | The diameter of a circle equal to the contour area |

**References**

[1] Justes E, Mary B, Meynard JM, Machet JM, Thelierhuche L. 1994. Determination of a critical nitrogen dilution curve for winter-wheat crops. Ann Bot. 1994;74:397–407.

[2] Tan W，Yang ZQ，Li J. Simulation of nutrient quality of Pakchoi based on temperature-light function. Chin J Agrometeorol. 2015;2:1234-1242. [In Chinese Language]

[3] Nunn C, Hastings AF, Kalinina O, Özgüven M, Schüle H, Tarakanov IG, Van Der Weijde T, Anisimov AA, Iqbal Y, Kiesel A, Khokhlov NF. Environmental influences on the growing season duration and ripening of diverse Miscanthus germplasm grown in six countries. Front. Plant Sci. 2017;8:907.

[4] Stockle C, Donatelli M, Nelson R. CropSyst, a cropping systems simulation model. Eur J Agron. 2003;18:289–307.

[5] Xiong X, Zhang JJ, Guo DD, Chang LY, Huang DF. Non-invasive sensing of nitrogen in plant using digital images and machine learning for brassica campestrisssp. chinensis L. Sensors. 2019;19:2448.
